# Deciphering the Draft Genome of *Toxoplasma gondii* RH Strain

**DOI:** 10.1371/journal.pone.0157901

**Published:** 2016-06-29

**Authors:** Yee-Ling Lau, Wenn-Chyau Lee, Ranganath Gudimella, GuiPing Zhang, Xiao-Teng Ching, Rozaimi Razali, Farhanah Aziz, Arif Anwar, Mun-Yik Fong

**Affiliations:** 1 Tropical Infectious Diseases Research and Education Centre (TIDREC), Department of Parasitology, Faculty of Medicine, University of Malaya, Kuala Lumpur, Malaysia; 2 Singapore Immunology Network (SIgN), Agency for Science, Technology and Research (A*STAR), Singapore, Singapore; 3 Sengenics HIR, University of Malaya, Kuala Lumpur, Malaysia; 4 BGI-Shenzhen, ShenZhen, China; NIH, UNITED STATES

## Abstract

Toxoplasmosis is a widespread parasitic infection by *Toxoplasma gondii*, a parasite with at least three distinct clonal lineages. This article reports the whole genome sequencing and *de novo* assembly of *T*. *gondii* RH (type I representative strain), as well as genome-wide comparison across major *T*. *gondii* lineages. Genomic DNA was extracted from tachyzoites of *T*. *gondii* RH strain and its identity was verified by PCR and LAMP. Subsequently, whole genome sequencing was performed, followed by sequence filtering, genome assembly, gene annotation assignments, clustering of gene orthologs and phylogenetic tree construction. Genome comparison was done with the already archived genomes of *T*. *gondii*. From this study, the genome size of *T*. *gondii* RH strain was found to be 69.35Mb, with a mean GC content of 52%. The genome shares high similarity to the archived genomes of *T*. *gondii* GT1, ME49 and VEG strains. Nevertheless, 111 genes were found to be unique to *T*. *gondii* RH strain. Importantly, unique genes annotated to functions that are potentially critical for *T*. *gondii* virulence were found, which may explain the unique phenotypes of this particular strain. This report complements the genomic archive of *T*. *gondii*. Data obtained from this study contribute to better understanding of *T*. *gondii* and serve as a reference for future studies on this parasite.

## Introduction

*Toxoplasma gondii* is a globally widespread zoonotic parasite that infects approximately one-third of the world's population [1, 2]. Although most *T*. *gondii* infections are subclinical, infection by this parasite in immuno-compromised groups and pregnant women can result in severe outcomes [3, 4]. As *T*. *gondii* can persist within the host via latent infection, it may cause catastrophic consequences to those infected with HIV following reactivation of the parasite. This parasite is incriminated as one of the most fatal foodborne pathogens in USA [5].

Similar to many other members of the Apicomplexa phylum, *T*. *gondii* shows a complex life cycle, during which sexual reproduction happens within its definite host (cat). Asexual reproduction occurs within its wide range of homoeothermic intermediate hosts, including humans. Since sexual reproduction is part of its life cycle, *T*. *gondii* was expected to show high variation across the world. However, the majority of the *T*. *gondii* strains from most parts of the world belong (but are not restricted) to three distinct clonal lineages (type I, type II and type III) with minimal genetic differences among them [6–8]. However, these *T*. *gondii* lineages show varied virulence [9]. For instance, Type I *T*. *gondii* is the most virulent lineage in murine models [10]. Furthermore, type I has been persistently and disproportionately isolated from immuno-competent patients suffering from severe ocular toxoplasmosis in USA [11]. Type I is also associated with severe congenital toxoplasmosis in European countries [10, 12].

The *T*. *gondii* type I lineage is represented by GT1 (isolated from goat’s skeletal muscle) and RH (isolated from a fatal toxoplasmosis-induced encephalitis case in year 1939) strains [13, 14]. RH strain has been adapted to *in vitro* cultivation and commonly used in laboratory work [15]. It shows high growth and migration rates, and imparts the highest fatality in mice [9, 16]. On the other hand, type II (represented by ME49 strain) and type III (represented by VEG and CEP strains) are considered less virulent than type I [17], with type II dominating human toxoplasmosis in USA [18, 19] while type III is significantly associated with animal hosts [18, 20]. With high genetic similarities between these lineages, it is intriguing to see apparently varied virulence among these parasites [9].

The mechanisms behind the virulence and pathogenesis as well as other biological aspects of *T*. *gondii* can be understood better by performing in-depth genome analyses and comparisons on all major lineages of *T*. *gondii*. To date, whole genome sequencing has been completed on 62 *T*. *gondii* strains, including type I (GT1 and RH strains), type II (ME49 strain) and type III (VEG strain), as well as recombinant strains [21–27]. A clearer picture on genome differences between the major *T*. *gondii* lineages will contribute towards better understanding on different virulence attributed to these lineages. Here, we performed whole genome *de novo* sequencing and assembly on *T*. *gondii* RH strain and compared its genome with those of *T*. *gondii* GT1, ME49 and VEG strains.

## Materials and Methods

### Ethical approval

Experiments involving mouse model were carried out in compliance with the animal ethics approved by Institutional Animal Care and Use Committee (IACUC) of the University of Malaya, Faculty of Medicine (2014-06-03/PARA/R/CXT).

### Propagation of parasites and genomic DNA extraction

Tachyzoites of *T*. *gondii* RH strain were maintained by serial intraperitoneal passage in BALB/c mice. The tachyzoites were harvested from peritoneal fluids three to four days-post- infection. The parasites were washed and re-suspended in sterile phosphate buffered saline (1x PBS) prior to usage. High molecular weight genomic DNA was extracted using DNeasy Blood and Tissue Kit (QIAGEN, Germany). The specific identity of the extracted DNA was verified by conventional nested PCR directed against the B1 gene of *T*. *gondii* (primers used: B1F1:5’-CCGTTGGTTCCGCCTCCTTC-3’; B1R1: 5’-GCAAAACAGCGGCAGCGTCT-3’ and B1F2: 5’-CCGCCTCCTTCGTCCGTCGT-3’; B1R2: 5’-GTGGGGGCGGACCTCTCTTG-3’) and Loop-Mediated Isothermal Amplification Method (LAMP) [28, 29]. The DNA yield and purity were measured spectrophotometrically (NanoDrop 2000c spectrophotometer, Thermo Scientific); DNA integrity was verified by agarose gel electrophoresis and a Bioanalyzer (2100, Agilent).

### Library construction and sequencing of *T*. *gondii*

DNA samples of *T*. *gondii* were used for the constructions of five libraries with various insert sizes of 200 bp, 500 bp, 800 bp, 5 kb and 10 kb. Paired-end sequencing was performed on these five libraries through Illumina HiSeq 2000 which allows users to determine the length of the insert and to sequence both ends of the insert, generating high quality sequence data. This process generated a total of 30.89 Gb of raw sequence data (Table A in S2 File).

### Sequence filtering

Artificial reads or low quality paired reads derived mainly from base-calling duplicates and adapter contamination were filtered to obtain a clean read set. Base-calling duplicates, which are pseudo-sequences caused by the SOLEXA pipeline, were filtered at a threshold of Euclidean distance ≤ 3 and a mismatch rate of ≤ 0.1. Duplicated reads from PCR amplification during library construction (both long inserts of ≥ 2 kb and short inserts of 150–500 bp) were filtered to ensure high accuracy in scaffold construction. Besides, removal of low quality sequences such as those with an excess of “N” bases (> 10%) was done. Different bacterial and viral genomes were used for similarity mapping with Burrows-Wheeler Aligner (BWA). Reads mapped to the bacterial and viral genomes were removed. From these steps, the data size of all five libraries (post-filtered) was reduced significantly (Table B in S2 File).

### Genome assembly

The sequencing errors were corrected based on *k*-mer frequency information before assembly in order to reduce the memory consumption in de Bruijn graph algorithm construction [30]. We had chosen 17-mers and corrected the sequencing errors for frequency lower than 4. The total used bases were 6.63 Gb and used reads were 66,335,501 whereas the specificity *k*-mer number was 231,186,702 (Table C in S2 File). The expected depth peak was 100 according to the distribution curve (Figure A in S1 File).

The corrected reads were assembled using SOAPdenovo, a software that assembles short oligonucleotide into contigs and scaffolds through de Bruijn graph algorithm [31]. During assembly, a de Bruijn graph was used to assemble all possible sequences from the Illumina reads, with a *k*-mer as a node and the k-1 bases overlap between two *k*-mers as an edge. In this *T*. *gondii* genome assembly, we chose *k* = 25 bp (25-mers) to construct de Bruijn graph. To avoid chimeric reads, which are responsible for misassembles by generating incorrect sequence overlap, only short insert-size (<1 kb) of single- and paired-end reads were used in this assembly. Further corrections performed during de Bruijn graph simplification encompassed errors removals (tips, low coverage linkages and bubbles removal) and tiny repeats resolving. The graph was transformed to a contig graph by transforming linearly connected *k*-mers into a pre-contig node. Dijkstra’s algorithm (Skiena) was used to detect bubbles, which were then merged into a single pathway when the branches sequences were identical. Using this method, repetitive sequences were collapsed and consensus sequences were obtained.

The contig sequences were obtained by conjoining the *k*-mers in an unambiguous path. These contigs were broken into fragments at the boundaries of repeat ambiguous connections. All reads were realigned onto the contig sequences to obtain aligned paired-end reads. Contigs were linked to a scaffolding graph with paired-end reads. Connections between contigs comprised the edges in this graph, and the branch length demonstrated the gap size, which was calculated from the insert size of the paired-end reads. Subsequently, sub-graph linearization was applied to transform interleaving contigs into a linear structure. Repeat masking was used to mask complicated connections for repeat contigs. Following this, the amount of shared paired-end relationships between contig pairs were calculated, the ratio of consistent and conflicting paired-ends were determined, and scaffolds construction was done from the shortest to the longest insert size paired-ends in a step by step manner.

Gaps, which composed mainly of masked repeats during scaffold construction, were found. Through the paired-end information, we retrieved read pairs that had one read well-aligned on the contigs and another read located in the gap region. Local assembly was performed on these reads to fill in small gaps within the scaffolds. We constructed a 27-mers de Bruijn graph with reads in the gaps and the contig ends on both sides using approach similar to that of contig construction. Gaps were filled with path sequences if an unambiguous path was found between those two contig ends. Each read in the gaps was checked to find one that had unambiguously mapped ends on both sides of the contigs and filled the gaps with read sequences. Most of the small tandem-repeat gaps were correctly filled by this way. However, longer tandem-repeat gaps could not be resolved by read sequences. Hence, we filled them with two repeat units along with a string of “N”. The GC-depth content difference is a primary factor for non-random sequencing-depth distribution [32]. Therefore it was examined to analyse the nucleotide distribution, to assess the randomness of sequencing and to inspect for possible sample contamination. Sliding of 500 bp bins (with 250 bp overlap) was applied for the examination.

### Repeats

Many assembled genomes consist of repeated sequences which have to be reduced to avoid misassembly and to minimize the error rate. A combination of homology-based and *de novo* approach was employed in this study to identify the interspersed repeated sequences. Known transposable elements (TEs) were identified using RepeatMasker and RepeatProteinMask against the Repbase library of TEs (Table D in S2 File) [33–35]. The RepeatProteinMask was used for identifying highly diverged TEs. On the other hand, we constructed a repeat library with RepeatModeler using two programs; RECON and RepeatScout for the *de novo* approach [36, 37]. The outputs were consensus sequences and classification information for each repeat family. Number of repeats was further identified through several tools such as DNA, SINE, LINE and LTR (Table E in S2 File).

### Gene annotation

Both homology-based and *de novo* approaches were employed in the predictions of gene structures in the draft genome assembly. Four gene sets from database ToxoDB (version 13.0) were used in homology-based approach [38]; *Neospora caninum*, *T*. *gondii* GT1, *T*. *gondii* VEG and *T*. *gondii* ME49, whereas *de novo* approach involved usage of AUGUSTUS, Genscan and SNAP softwares to filter partial and small genes with coding length shorter than 150 bp in order to reduce false positives. The predictions were aligned to a TE protein database using BlastP with E-value of 10^−5^ and filtered TE-derived genes that had more than 50% aligning rate. Different gene sources were then integrated to form a consensus gene set using GLEAN [39] by combining results from different gene predictions into a single set of gene prediction (Tables F and G in S2 File). Subsequently, individual genes were assigned functional annotation based on clustering Method. Functional annotation of the unique *T*. *gondii* genes has revealed key data that can further our understanding of differences between different strains. Uniprot database [40] was referred to understand the structure, subcellular location and functions of annotated proteins.

Besides, whole genome synteny analysis was performed. Based on the chromosomes of *T*. *gondii* GT1 strain, contigs reordering was done on the assembled scaffolds of *T*. *gondii* RH strain, where all the 14 chromosomes were reordered and reassembled with Mauve Aligner [41]. Similarities in chromosomes across different strains of *T*. *gondii* were studied using BLASTn (E value < 1e-05). Synteny blocks were retrived from the blast results to constitute the synteny plot with Circos v0.69 [42].

### Clustering of gene orthologs

All protein sequences were compared against a database containing a protein dataset of all the species studied (*T*. *gondii* RH, *T*. *gondii* GT1, *T*. *gondii* ME49, *T*. *gondii* VEG, *Neospora caninum*) (Table H in S2 File) (BlastP; using an E-value < 10^−7^), and con-joined fragment alignments for every gene were used through program Solar (Skiena). A connection (edge) was assigned between two nodes (genes) if more than one third of the region aligned to both genes. A h-score (0 to 100) was used to weight the similarity (edge). For two genes, G1 and G2, the h-score was defined as score (G1xG2) / max score (G1G1), score (G2G2), whereby the score was actually the BLAST raw score. Extracting gene families using clustering by Hcluster_sg, the average distance were used for the hierarchical clustering algorithm, requiring the minimum edge weight (h-score) to be larger than five, and minimum edge density (total number of edges / theoretical number of edges) to be larger than one third. The clustering of gene families was terminated if out-group genes were identified.

### Phylogenetic reconstruction

Protein sequences of parasite gene families between *T*. *gondii* strains were retrieved from previous work on *T*. *gondii* genomes [27] to run multiple sequence alignment using MAFFT v7 program (default option) [43]. Phylogeny tree editing and annotations were performed on iTOL webserver [44]. Phylogeny tree was constructed using Neighbour joining algorithm in Mafft online server [45].

### Data Access

This Whole Genome Shotgun project has been deposited at DDBJ/EMBL/GenBank under the project accession LLKL01000000 (BioProject number, PRJNA294483; BioSample, SAMN04026592), consists of sequences LLKL01000001-LLKL01000441.

## Results

### Genome assembly and repeat content

The genome of *T*. *gondii* RH strain (TOXRH) was sequenced at 171-fold coverage (Table 1). The total *k*-mer number was 5,402,162,948 with the calculated depth peak of 86 (Table C in S2 File). A final draft assembly of 441 scaffolds totalling 69.35 Mb in length was produced (Table 1). All gaps in RH strain assembled genome were filled similarly to GT1 and VEG strains. Scaffolds with length shorter than 200 bp were excluded (Table I in S2 File). Besides, chromosome assemblies were conducted (Table J in S2 File).

**Table 1:** Comparison of assembly statistics. ToxoDB version 13.0 was referred.

Details	*T*. *gondii* RH	*T*. *gondii* GT1	*T*. *gondii* ME49	*T*. *gondii* VEG	*Neospora caninum*
**Coverage (x)**	171	67.44	26.5	77.38	N/A
**Genome size (Mb)**	69.35	63.95	65.67	64.52	59.1
**Number of Scaffolds**	441	2063	2263	1323	66
**Contig**	7797	2337	1243	1340	52
**Scaffolds N50 (kb)**	2839.2	6105.4	6327.7	6372.9	5490.9
**GC (%)**	52.0	52.4	52.3	52.4	54.8
**Coding genes (n)**	7860	8460	8322	8410	7122
**Chromosomes**	14	14	14	14	14

The majority of the sequences have a GC content between 40–60% (mean = 52%) and almost all have a sequencing depth above 60X (Figures B-D in S1 File). This is indeed similar to *T*. *gondii* GT1 (Type I), *T*. *gondii* ME49 (Type II) and *T*. *gondii* VEG (Type III) (Figure C in S1 File). We detected 190 (76.61%) core essential genes by CEGMA, indicating that the assembly represents a substantial proportion of the entire genome. We also estimated a repeat content of 15.98% (equating to 11.15 Mb of DNA) in this draft genome (Table D in S2 File), comprised of 5.136% (3,583,811 bp) DNA transposons, 1.735% (1,210,317 bp) LINE, 0.037% (25,965 bp) SINE, 4.858% (3,389,530 bp) LTR and 2.914% (2,033,436 bp) unclassified dispersed elements (Table E in S2 File). Additionally, we have estimated segmental duplication of 1.69 Mb.

### Gene set comparison of *T*. *gondii* RH strain with *T*. *gondii* GT1, ME49 and VEG strains

**Fig 1:**
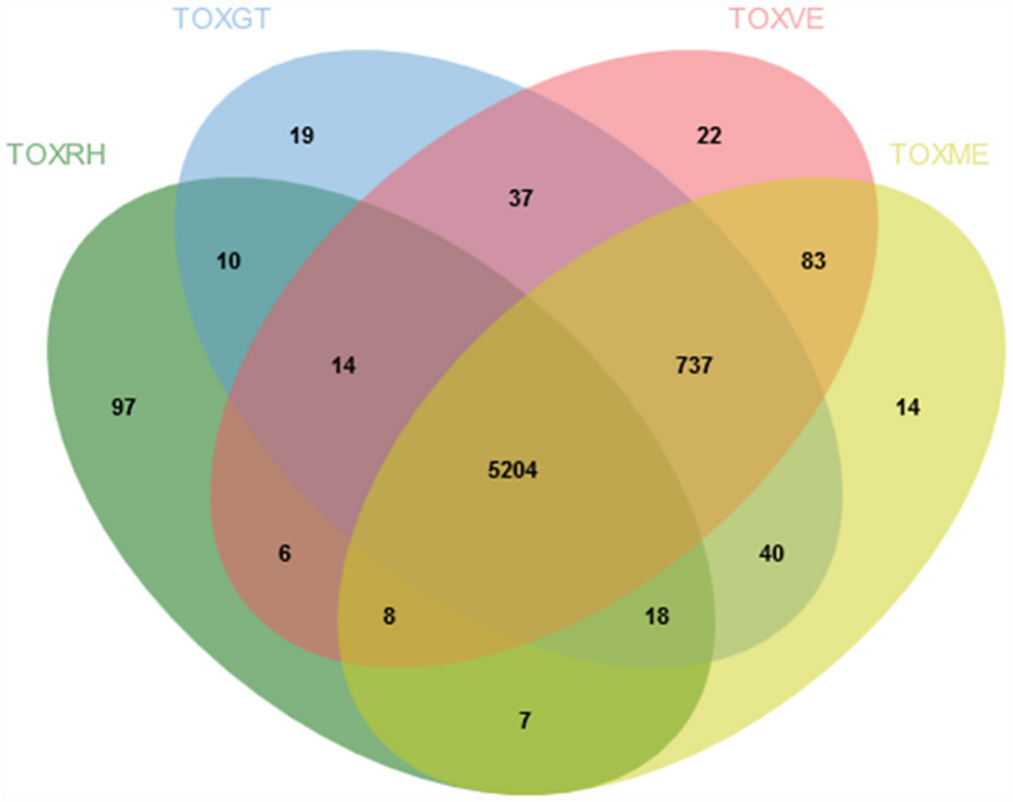
Genome comparison of *T*. *gondii* RH, GT1, ME49 and VEG strains. Abbreviation: TOXME: *T*. *gondii* ME49 strain; TOXVE: *T*. *gondii* VEG strain; TOXGT: *T*. *gondii* GT1 strain; TOXRH: *T*. *gondii* RH strain.

**Fig 2:**
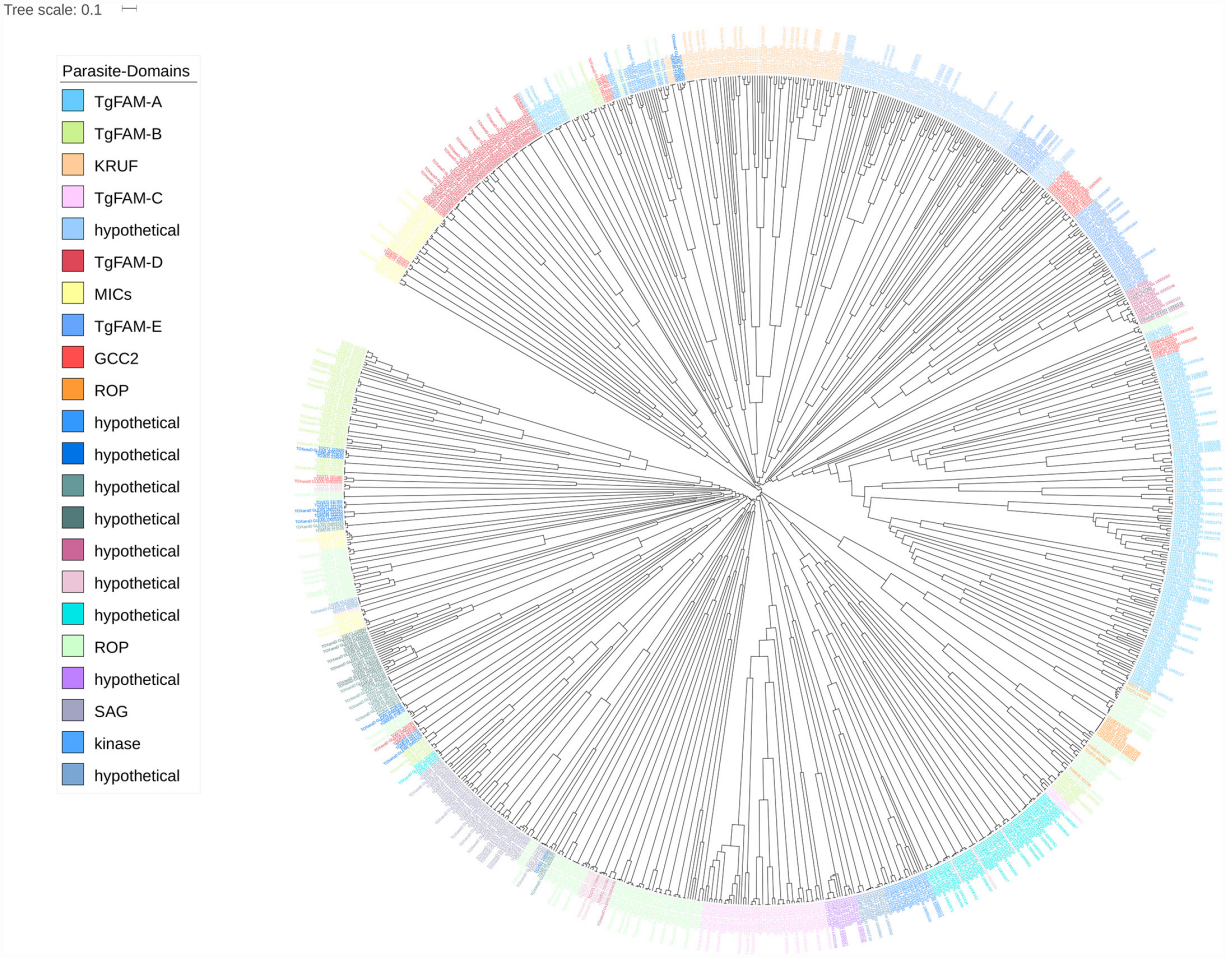
Phylogeny tree in circular format representing genes related to the parasite gene families of *T*. *gondii*. Different colors represent the various parasite gene families.

**Fig 3:**
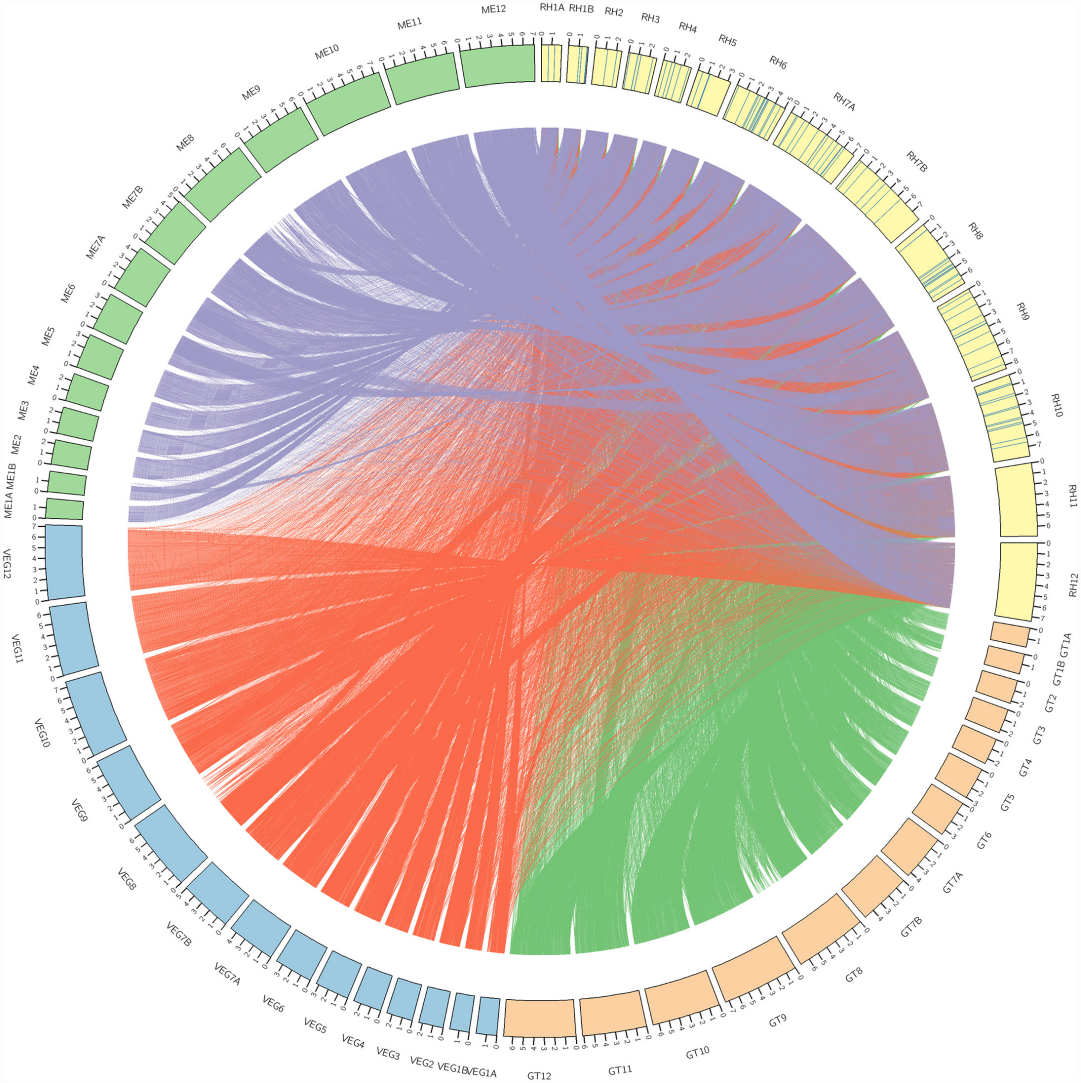
Circos plot illustrating the levels of synteny among genomes of different *T*. *gondii* strains. Outer ring represents chromosomes of each strain, with the labelling of respective chromosome numbering. The scale marks on the chromosome represent 1Mb. The color bands represent syntenic blocks between the chromosomes under comparison with the chromosomes of *T*. *gondii* RH. The blue lines within the chromosome blocks of *T*. *gondii* RH indicate the *T*. *gondii* RH-unique genes identified as part of the orthologous gene study.

GLEAN analysis predicted 7860 protein-encoding genes and 216 non-coding DNAs. The average length of exons and introns are 419 bp and 548 bp, respectively, with an average of 5.9 exons per gene (Table F in S2 File). This finding is similar to genomes of other *T*. *gondii* strains. Based on comparison with the draft genomes of *N*. *caninum*, *T*. *gondii* GT1, *T*. *gondii* ME49 and *T*. *gondii* VEG [38], *T*. *gondii* RH strain shows high sequence similarity to all three distinct strains of *T*. *gondii*. By applying the clustering method, gene families (homologous genes clustered together from different genomes) were analyzed. Of the predicted *T*. *gondii* RH gene families, 97.8% (5246 gene families) have orthologs (BLASTp cut-off: 10^−5^) in *T*. *gondii* GT1 compared to that in *T*. *gondii* ME49 (n = 5237; 97.6%) and *T*. *gondii* VEG (n = 5232; 97.5%) (Fig 1). From phylogenetic analysis (Fig 2), close evolutionary relationship between *T*. *gondii* RH and *T*. *gondii* GT1 was noted. This was in parallel with the results from the clustering method mentioned earlier. Both sets of results reflected sharing of a more recent common ancestor between *T*. *gondii* RH and *T*. *gondii* GT1. Between *T*. *gondii* ME49 and VEG strains, *T*. *gondii* RH showed closer relationship with *T*. *gondii* ME49. Meanwhile, 97 gene families were predicted to be unique to the *T*. *gondii* RH genome (Fig 1; Table H in S2 File). From the synteny analysis, we found that all three genomes of *T*. *gondii* GT1, ME49 and VEG showed high level of synteny with the genome of *T*. *gondii* RH. The generated synteny blocks reflected high chromosomal conservation between *T*. *gondii* RH and *T*. *gondii* GT1 whereas both *T*. *gondii* ME49 and *T*. *gondii* VEG showed smaller length chromosomal rearrangements. Generally, *T*. *gondii* RH has 99% similarity with all strains of *T*. *gondii* at chromosomal synteny level (Fig 3).

### Annotation and protein classifications

Three types of noncoding RNA were found, i.e., 181 copies of tRNA, 18 copies of rRNA and 4 copies of miRNA (Table K in S2 File). In all, 7563 genes were clustered in 5364 families with 97 unique families encoding a total of 111 unique genes (Tables H and L in S2 File). The unique genes found were indicated in the circos plot of synteny study as well (Fig 3). These unique genes were found to be distributed unevenly across all chromosomes of *T*. *gondii* RH except chromosomes 11 and 12 (Table M in S2 File). The *T*. *gondii* RH-unique genes were “concentrated” in chromosomes 8, 6, 10, 7A, and 9.

To understand more about evolutionary origin of the 111 *T*. *gondii* RH unique genes, we decided to conduct a protein-protein BLAST (BLASTp) screening against genomes of other *T*. *gondii* strains available from ToxoDB. From this local alignment, 12 of the 111 genes showed good BLAST matches with genes of other *T*. *gondii* strains, validating the presence of similar homologs within the genomes of other *T*. *gondii* strains (Table N in S2 File). BLASTX of *T*. *gondii* ME49 unique genes found previously [27] against the unique genes found in *T*. *gondii* RH draft genome was performed. There were nine genes with unknown annotations showing good matches (Table O in S2 File). Besides, unique gene synteny similarity search between the *T*. *gondii* RH scaffold and *T*. *gondii* GT1 chromosomes were conducted. From this investigation, only eight of the *T*. *gondii* RH unique genes showed overlaps of synteny and the remaining 103 genes were specific to *T*. *gondii* RH strain (Table P in S2 File). These findings suggest divergence evolution of *T*. *gondii* strains from a common ancestor, giving rise to the many *T*. *gondii* RH genes that are not recognizable as homologs under comparisons with genomes of other *T*. *gondii* strains.

Most of the unique genes were annotated to unknown functions. Nevertheless, 17 unique genes were annotated to proteins with vital functions (Table Q in S2 File). Functions annotated by the 11 genes involved in gene expression regulation could be classified into pre-translational regulation (TOXaeaD_GLEAN_10004604, TOXaeaD_GLEAN_10007767, TOXaeaD_GLEAN_10006721, TOXaeaD_GLEAN_10006347), translation “patrolling” (TOXaeaD_GLEAN_10005699, TOXaeaD_GLEAN_10005558), and post-translational modifications (TOXaeaD_GLEAN_10000645, TOXaeaD_GLEAN_10000683, TOXaeaD_GLEAN_10003567, TOXaeaD_GLEAN_10005019, TOXaeaD_GLEAN_10005632). Differences in regulation of gene expression may lead to different and unique phenotypes. Importantly, these genes may play critical roles in epigenetics of the *T*. *gondii* RH strain, which may contribute to distinct phenotypes among different strains despite high similarity at the genomic level. Indeed, epigenetics of *T*. *gondii* have received more research attention in the field of parasitology [46]. In addition, *T*. *gondii* is known for its temporal regulation of gene expression in conjunction with stage transition [47]. When compared with the type I and type II parasites, *T*. *gondii* RH strain has relatively lower tendency to form bradyzoites. These *T*. *gondii* RH-unique genes may involve in its regulation of stage transformation (i.e., tachyzoite to bradyzoite) that is different from other *T*. *gondii* clonal lineages.

Four *T*. *gondii* RH unique genes were annotated to regulation of replication, encompassing DNA repairing (TOXaeaD_GLEAN_10004705), mitotic cell cycle regulation (TOXaeaD_GLEAN_10005699, TOXaeaD_GLEAN_10006721, TOXaeaD_GLEAN_10004705), and meiosis regulation (TOXaeaD_GLEAN_10004604). It is noteworthy that the unique genes responsible for replication regulation were found to be annotated to functions that are closely associated with gene expression regulation as well. After all, the speed of replication is linked to the tachyzoite-bradyzoite stage conversion [48], which implies different protein expressions.

Two unique genes were annotated to exosomal proteins carrying calcium-related functions. Gene TOXaeaD_GLEAN_10001862 was annotated to hippocalcin-like protein 1 (HPCAL1). As indicated by the name, this protein is involved in calcium ion binding. Another unique gene (TOXaeaD_GLEAN_10003157) is annotated to calmodulin-like protein 3 (CALML3), which is also known as protein NB-1. CALML3 is an exosomal protein involved in calcium binding and release [49]. We believe that this protein may be involved in regulation of locomotion, invasion and egression [50, 51]. We have verified the presence of this gene within the *T*. *gondii* RH genome with PCR and sequencing as well (Figure E in S1 File). Needless to say, further work is needed to verify this postulation.

Two genes were found to be annotated to enzymatic digestion. Gene TOXaeaD_GLEAN_10006826 was annotated to pepsin II-4 (pepsin A), whereas gene TOXaeaD_GLEAN_10004048 was annotated to a putative peroxisomal acyl-coenzyme A oxidase 1.2, which catalyzes the desaturation of acyl-CoAs to 2-trans-enoyl-CoAs. It regulates beta oxidation of fatty acids, which transforms lipids into acetyl CoA. The transformed acetyl CoA will then be led into Kreb’s cycle for energy production. Efficient metabolism and energy generation may enable *T*. *gondii* RH strain to stay “active” as tachyzoites instead of facing “starvation” that drives bradyzoite stage conversion [52].

One gene (TOXaeaD_GLEAN_10002720) was annotated to a transporter known as multidrug resistance-associated protein 1 (MRP1), which is also known as ATP-binding cassette sub-family C member 1 protein (ABC.C1). The ABC proteins have been linked to drug resistance in protozoan parasites [53]. This “detox” transporter acts as a multi-specific organic anion transporter, with oxidized glutathione, cysteinyl leukotrienes and activated aflatoxin B1 as substrates. It also transports glucuronides and sulfated conjugates of steroids and bile salts. Indeed, it was reported in an earlier study that three ABC transporters of *T*. *gondii* RH (which showed larger plaque formation) were significantly over-expressed when compared to *T*. *gondii* GT1, suggestive of association between the ABC transporters and the parasite’s higher growth rate [54]. Besides, a recent study found that the ABC.C1 of type I *T*. *gondii* is different from those of non-type I *T*. *gondii* parasites [55]. Although sulfadiazine resistance in *T*. *gondii* has been shown to be unrelated to ABC transporters [55], we believe that this *T*. *gondii* RH-unique ABC transporter may play important roles in detox machinery of the parasite. This protein may modulate accumulation and removal of other toxic substances or xenobiotics, which enables longer extracellular survival for the parasite.

## Discussion

Whole genome alignments represent the fundamental basis for comparative analyses aimed at identifying and characterising functional related stretches of genes that are clustered. For instance, similarity across a wide range of evolutionary distance can be detected by a multiple alignments of homologous sequences from several species, whereby conserved and important biological similarities are usually revealed. Similarly, estimation of local rates of evolution on the basis of multiple alignments provides quantitative assessment of the strength of evolutionary constraints and the importance of functional elements. Collectively, these contribute to better understanding of the cellular biology of a parasite.

In this study, we generated a draft genome of *T*. *gondii* RH strain that was slightly larger than those reported by a number of studies [22–24, 27]. This may be due to a number of reasons. The performed sequencing depth of coverage is one of the possible reasons. Increased depth of coverage can compensate the possible presence of sequencing errors in the short reads, which may otherwise be filtered even if the reads are not sequencing errors. Thus, more reads are retained, resulting in a larger assembly. Besides, the larger amount of non-coding DNAs within the draft genome of *T*. *gondii* RH strain used in this study may contribute to the larger genome size as well. Interestingly, the number of coding genes in *T*. *gondii* RH draft genome of this study happened to be of the lowest under comparison with the genomes of *T*. *gondii* GT1, ME49 and VEG strains (Table 1). Nevertheless, the draft genome size predicted from this study is within the expected size range [56].

By referring to a list of pathogenesis-associated, parasite gene families presented by a recently published work [27], we constructed a phylogeny tree to study the evolutionary relationship between *T*. *gondii* RH, GT1, ME49 and VEG strains. We managed to find 23 of the 24 reported parasite-specific domains. Only one domain (para_38; with repeat motifs) was not found in *T*. *gondii* RH draft genome (Table R in S2 File). A total of 1041 genes from the genome of *T*. *gondii* RH, and the published genomes of *T*. *gondii* GT1, VEG and ME49 strains were recruited. Interestingly, *T*. *gondii* RH genome had the smallest number of genes in most of these gene families as compared with the GT1, VEG and ME49 strains. *T*. *gondii* RH draft genome assembled from this study had more genes in few of the domains (Para_101 and Para_27, both annotated to hypothetical proteins; and Para_41 and Para-44, which were annotated to rhoptry proteins) than draft genomes of other strains. Such differences may be associated with certain phenotypic variations among the different strains of *T*. *gondii*. On the whole, *T*. *gondii* RH shared the closest evolutionary relationship with *T*. *gondii* GT1, as compared with the ME49 and VEG strains.

Our genome synteny analysis revealed that *T*. *gondii* RH genome shared high level of synteny with the genomes of three strains of *T*. *gondii* (GT1, ME49 and VEG) (Fig 3). The level of synteny depends on chromosomal rearrangement throughout the process of evolution. Chromosomal rearrangement can happen via chromosome translocation, inversion, fission and fusion. These processes result in loss of synteny between chromosomes under comparison. Likewise, such rearrangement may restore the synteny between chromosomes under comparison along the course of evolution. Previously, it was suggested that crossing of type II *T*. *gondii* with other ancestral strains gave rise to type III (as the first generation offspring) and type I (likely the second generation offspring) parasites [57]. It is likely that the incidence of chromosomal rearrangement was low along the course of evolution from type II to type III and type I parasites.

Different strains of *T*. *gondii* show varied biological properties. For instance, type I strain was reported to show higher trans-epithelial migration rate than type II and type III strains [58]. Indeed, even *T*. *gondii* strains of the same clonal lineage have been reported to demonstrate different phenotypes. The laboratory-adapted RH strains are known to be incapable of forming bradyzoites that are responsible for chronic infection [59]. Furthermore, the RH strain also poses a higher extracellular survival rate than GT1 strain [54]. These biological phenotype variations contribute to the virulence differences among these parasites. Intriguingly, the three major *T*. *gondii* clonal lineages show high similarity at the genome level, with the RH strain having only 111 unique genes. Therefore, these unique genes may be responsible for the different characteristics of *T*. *gondii* RH strain compared to other strains. We managed to locate these genes at the chromosomes of the parasite (Fig 3 and Table M in S2 File). Only chromosomes 11 and 12 did not have any *T*. *gondii* RH- unique genes. Most of these 111 *T*. *gondii* RH-unique genes were annotated to proteins with unknown functions. Nonetheless, 17 genes were annotated to proteins known for functions that are vital to cellular biology, many of which were related to regulation of gene expression.

By analyzing *T*. *gondii* RH draft genome, we managed to understand better the genomic structure and evolution of this strain. The differences between the closely related *T*. *gondii* genomes were studied. We unraveled a short list of genes unique to *T*. *gondii* RH strain, where annotation of a number of unique genes fits in elegantly with findings from previous *T*. *gondii* RH genome-wise studies.

The genome of *T*. *gondii* RH was recently archived [27]. Nevertheless, we did not do comparison between the published *T*. *gondii* RH draft genome and the *T*. *gondii* RH draft genome assembled from this study. As the recently published *T*. *gondii* RH genome is a raw data without assemblies, any comparison done with such raw data would be incomplete. Needless to say, the availability of a richer *T*. *gondii* genomic database contributed by different parties will definitely benefit researchers in future studies.

The unique genes described above may be the genetic markers responsible for phenotypic differences between *T*. *gondii* RH strain and other strains of *T*. *gondii*. Nevertheless, it is important to acknowledge the difficulty of drawing a solid conclusion with only data garnered from draft genomes. Genomic comparison would be more powerful with downstream experiments such as transcriptome comparisons, gene silencing, gene knock-out and knock-in experiments. However, our findings have highlighted a short list of potential gene candidates, which may serve as a reference for future in-depth studies. Further investigations on the *T*. *gondii* RH unique genes with hypothetical protein annotations may complement our understandings on the virulence difference among the different strains of *T*. *gondii*. This genome is useful as a resource to study gene rearrangements of *T*. *gondii* as well.

## Conclusions

We have performed full genome sequencing and *de novo* assembly on the RH strain of *T*. *gondii*, which complement the genomic archive of *T*. *gondii*. High genome similarity between the different strains of *T*. *gondii* was seen, and 111 *T*. *gondii* RH strain-unique genes were found, some of which may be related to the distinct phenotypes of this particular strain. Data obtained from this study contribute to better understanding on *T*. *gondii* and serve as a reference for future studies on this parasite.

## Supporting Information

S1 File **Figure A. Estimation of genome size of *T*. *gondii* RH strain using K-mer analysis. Figure B. GC content and sequencing depth of the *T*. *gondii* RH strain recruited in this project. Figure C. GC content distributions for the genome**. The x-axis represents GC content and the y-axis represents the proportion of the bins number divided by the total windows. **Figure D. Sequence depth distribution curve. Figure E. Primer design, PCR and sequencing of a randomly selected *T*. *gondii* RH-unique gene (TOXaeaD_GLEAN_10003157) found in this study**.
(DOC)

S2 File Table A. Statistic of pre-filter data. Table B. Statistic of post-filter data. Table C. 17-mer statistics information. Table D. General statistics of repeats in genome. Table E. TEs content in the assembled *T*. *gondii* RH genome. Table F. General statistics of predicted protein-coding genes. Table G. Summary of evidence for the GLEAN gene models. Table H. Statistical analysis of gene families. Table I. Statistics of the assembled sequence length. Table J. Chromosome assembly. Table K. Non-coding RNA genes in the genome. Table L. The 111 genes were predicted to be unique to the *T*. *gondii* RH genome based on Clustering Method. Table M. Chromosomal loci of the 111 *T*. *gondii* RH-unique genes. Table N. BLASTp result of 111 unique genes to the latest release from Toxodb.org. Table O. BLASTx of *T*. *gondii* ME49 unique genes vs *T*.*gondii* RH (external data retrieved: supplementary table G of Lorenzi *et al*. 2016[27]). Table P. Synteny overlaps of the *T*. *gondii* RH unique gene loci on scaffolds with the *T*. *gondii* GT1 genome. Table Q. The 17 unique genes were annotated to proteins with vital functions. Table R. Single Copy clusters from *T*. *gondii* RH comparad to recently published gene families of *T*. *gondii* containing unique parasite-specific (para) domains. (external data retrieved: supplementary table G of Lorenzi *et al*. 2016[27]).
(XLS)
