# Supplementary material for: Deciphering the Draft Genome of Toxoplasma gondii RH Strain
Source: PLoS One. 2016 Jun 29;11(6):e0157901. doi: 10.1371/journal.pone.0157901 (PMC4927122; doi:10.1371/journal.pone.0157901)
Supplement: S1 File — Figure A. Estimation of genome size of T. gondii RH strain using K-mer analysis. Figure B. GC content and sequencing depth of the T. gondii RH strain recruited in this project. Figure C. GC content distributions for the genome. The x-axis represents GC content and the y-axis represents the proportion of the bins number divided by the total windows. Figure D. Sequence depth distribution curve. Figure E. Primer design, PCR and sequencing of a randomly selected T. gondii RH-unique gene (TOXaeaD_GLEAN_10003157) found in this study. (DOC) [file pone.0157901.s001.doc]

**S1 File**

**Figure A**

**Figure B**

**Figure C**

**Figure D**

**Figure E**

**TOXaeaD_GLEAN_10003157
Calmodulin-like protein 3 (CaM-like protein) (CLP) (Calmodulin-related protein NB-1)**>TOXaeaD_GLEAN_10003157 (438 bp)

ATGCATGCCACAGGGTGTTTTCCCCGTAAAACACAGAGTTGCAGGTACTCTTGTCGTAATGTGTCGTGCAGAGATTTCGGGAAAATTTTCCGGTCCGTAGGTCAGAACCCTTCAGAGGAAACAATCCGCCAGTTAATTCAAATGTATGACGAGCAGAAGGGAGAAGGGCAGTTCAGCTTCACGGAGTTTCTGAGGATTTGTGAATCTCCTCACTTTCAAGATCCGATGAAAGAGGAAAAAGTTCTCGAGTCTTTCAGAGAATTCGACAAAGACGCAACGGACACAATCACATTACTGGAGCTCCGGTACATTCTACAGCAGCTAGGAGAACGTCTGTCCGACGATGAAGCAGATGAGTTCATCGAATGGGCTCAGAAGGTAGGCATTCTTTCAAGCACGTACGATACAGCTGTGACTGTTCTCTACCTTGGGCCTTAA

**Primers designed:**

Forward: 5’-GAT GCT CAT GCA TGC CAC AGG GTG TTT-3’

Reverse: 5’-CGT CAG CTT AAG GCC CAA GGT AGA GAA-3’

Expected PCR product size: 438 bp

**PCR conditions:**

PCR amplification was performed with the following thermal cycling conditions:

94˚C for 5 min, 35 cycles of 94˚C for 45 s, 59.6˚C for 30 s and 72˚C for 1min followed by final extension at 72˚C for 10 min.

Gel electrophoresis: Agarose 1% gel, 100 V for 30 min; ladder used: O’GeneRuler™

**PCR results:**


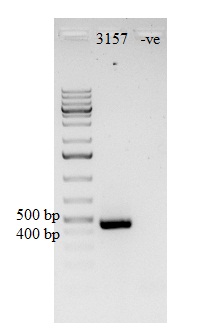


**Sequencing results:**

>160330Q5_A06_3157_3157F (398bp)

CAGAGTTGCAGGTACTCTTGTCGTAATGTGTCGTGCAGAGATTTCGGGAAAATTTTCCGGTCCGTAGGTCAGAACCCTTCAGAGGAAACAATCCGCCAGTTAATTCAAATGTATGACGAGCAGAAGGGAGAAGGGCAGTTCAGCTTCACGGAGTTTCTGAGGATTTGTGAATCTCCTCACTTTCAAGATCCGATGAAAGAGGAAAAAGTTCTCGAGTCTTTCAGAGAATTCGACAAAGACGCAACGGACACAATCACATTACTGGAGCTCCGGTACATTCTACAGCAGCTAGGAGAACGTCTGTCCGACGATGAAGCAGATGAGTTCATCGAATGGGCTCAGAAGGTAGGCATTCTTTCAAGCACGTACGATACAGCTGTGACTGTTCTCTACCTTGG

**PCR product Blast results:**

*T. gondii* ME49 calmodulin, putative, mRNA;

Sequence ID: XM_002371147.1

Length: 468 bp

Identities: 344/346 (99%)

*T. gondii* RH genome 160330Q5_A06_3157_3157F

Sequence ID: Query_193693

Length: 398 bp

Identities: 398/398 (100%)
